# Generation of functional oligopeptides that promote osteogenesis based on unsupervised deep learning of protein IDRs

**DOI:** 10.1038/s41413-022-00193-1

**Published:** 2022-03-01

**Authors:** Mingxiang Cai, Baichuan Xiao, Fujun Jin, Xiaopeng Xu, Yuwei Hua, Junhui Li, Pingping Niu, Meijing Liu, Jiaqi Wu, Rui Yue, Yong Zhang, Zuolin Wang, Yongbiao Zhang, Xiaogang Wang, Yao Sun

**Affiliations:** 1 Department of Oral Implantology, School of Stomatology, Tongji University, Shanghai Engineering Research Center of Tooth Restoration and Regeneration, Shanghai, 200072 China; 2 The First Affiliated Hospital of Jinan University, School of Stomatology, Clinical Research Platform for Interdiscipline of Stomatology, Jinan University, Guangzhou, 510630 China; 3 Key Laboratory of Big Data-Based Precision Medicine, School of Engineering Medicine, Beihang University, Beijing, 100191 China; 4 Guangzhou Laboratory, Bioland Laboratory, Guangzhou Regenerative Medicine and Health Guangdong Laboratory, Guangzhou, 510320 China; 5 Institute for Regenerative Medicine, Shanghai East Hospital, Shanghai Key Laboratory of Signaling and Disease Research, Frontier Science Center for Stem Cell Research, School of Life Sciences and Technology, Tongji University, Shanghai, 200092 China

**Keywords:** Metabolic bone disease, Bone

## Abstract

Deep learning (DL) is currently revolutionizing peptide drug development due to both computational advances and the substantial recent expansion of digitized biological data. However, progress in oligopeptide drug development has been limited, likely due to the lack of suitable datasets and difficulty in identifying informative features to use as inputs for DL models. Here, we utilized an unsupervised deep learning model to learn a semantic pattern based on the intrinsically disordered regions of ~171 known osteogenic proteins. Subsequently, oligopeptides were generated from this semantic pattern based on Monte Carlo simulation, followed by in vivo functional characterization. A five amino acid oligopeptide (AIB5P) had strong bone-formation-promoting effects, as determined in multiple mouse models (e.g., osteoporosis, fracture, and osseointegration of implants). Mechanistically, we showed that AIB5P promotes osteogenesis by binding to the integrin α5 subunit and thereby activating FAK signaling. In summary, we successfully established an oligopeptide discovery strategy based on a DL model and demonstrated its utility from cytological screening to animal experimental verification.

## Introduction

Peptide drugs are known to be highly selective, efficacious, and well tolerated by patients,^1^ and very short peptide drugs (oligopeptides) have been attracting increasing attention because of their high bioavailability and low cost of synthesis.^2–4^ A variant of the artificial intelligence method has been harnessed to substantially increase the efficiency of peptide drug development efforts^5^; these gains have been enabled by the abundant databases of available protein sequence and spatial structural information.^6^ However, these methods have been less impactful in the field of oligopeptide drug development due to issues including the relatively small amount of available data for oligopeptide drugs and the fact that the very short lengths of oligopeptides result in relatively few of the distinguishable features that are exploited by common machine learning approaches.^7^ Thus, the development of an automatic design strategy for oligopeptide drugs would be useful.

A report by Stavros et al. explored the interesting concept of the “no free lunch” theorem, which may yield insights that can help overcome the present shortage of available datasets for functional oligopeptides.^8^ For an increased amount of prior information to support function-related inferences—and perhaps even enhance the probability of successful drug discovery for oligopeptides—mining could be performed based on functional subsequences from a set of proteins with known functions related to a given process of interest. For example, cardiology researchers interested in identifying functional peptide sequences may be well served by narrowing their attention (search space) to the subset of proteins with functions in cardiogenesis. Extending this line of speculation, considering that many proteins contain intrinsically disordered regions (IDRs) that lack obvious structural features, we narrowed the range of protein sequences for data mining to the IDR. Recent studies have begun to reveal the biological functions of many IDRs, and there are now many examples wherein the interactions between IDRs and their target molecules are mediated by certain peptide motifs.^9^ Notably, many of these IDR-resident motifs are <10 residues in length.^10^ However, whether IDR sequences can be applied in the development of functional oligopeptides remains unknown.

In the present study, we explored the idea of using IDRs from a subset of process-specific proteins as a potential dataset for deep-learning-based identification of functional oligopeptides. Given the difficulty of identifying potentially informative features for oligopeptides, we envisioned that a natural language processing (NLP) model—which would have no need for manual prioritization of such features—would be a suitable approach to explore IDRs as a dataset for oligopeptide mining and functional prediction/elaboration. We elected to start our explorations using a basic N-gram model for inferring the semantic patterns of the IDRs from subsets of process-specific proteins to develop functional oligopeptide drug candidates. We focused on osteogenesis and used a simple model comprising an N-gram analysis of the predicted IDRs from 171 osteogenesis-annotated proteins and a Monte Carlo simulation to elaborate candidate 10-mer oligopeptides. After in silico evaluations, we synthesized and tested 28 candidate osteogenic oligopeptides, many of which exerted the anticipated effects in assays with bone marrow stem cells. One of these (AIB5P) effectively promoted osteogenesis both in vitro and in vivo. Ultimately, we experimentally characterized AIB5P’s osteogenic function: it binds to the integrin α5 subunit and activates FAK signaling, and we showed AIB5P’s promising therapeutic effects in studies with models of osteoporosis, fracture healing, and implant osseointegration.

## Results

### A deep learning-based strategy to screen for functional oligopeptides

**Fig. 1:**
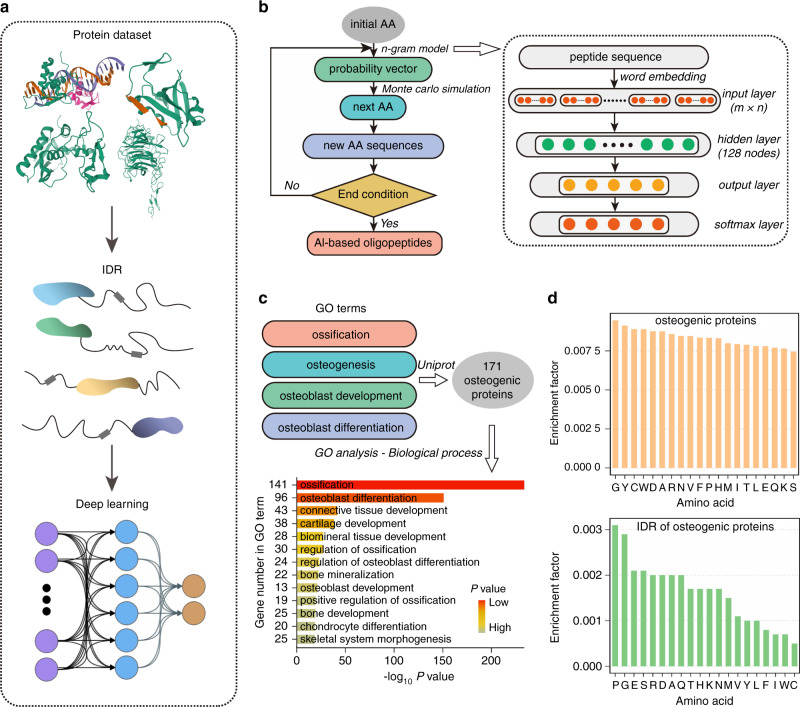
Design for a functional oligopeptide screening strategy using deep learning. **a** Workflow of the oligopeptide screening procedure. Briefly, the intrinsically disordered regions (IDRs) of proteins were employed to build a functional peptide motif dataset. **b** Architecture of the deep learning-based oligopeptide generation model: the model comprises two submodels, namely, N-grams and Monte Carlo simulation. The ten amino acids (AAs) with the highest frequency are used as the “initial AAs”. N-grams are used to infer the conditional probability of the context words (residues) of the oligopeptides awaiting extension, and the Monte Carlo simulation then generates the extended oligopeptides according to inferred probabilities. The overall model represents a repeating cycle of this basic process, with one AA extended in each cycle. The new oligopeptides obtained in each cycle then serve as oligopeptides awaiting extension in the next cycle until the end condition of the cycle is reached (here, until the oligopeptides have been extended to a total of 10 residues). **c** Protein candidates were retrieved from UniProt based on their reported involvement in bone formation by using four osteogenic GO terms: “ossification”, “osteogenesis”, “osteoblast development”, and “osteoblast differentiation”. A total of 171 protein candidates were thus collected. **d** Display of amino acid frequency distribution in the intrinsically disordered regions or full-length sequences of 171 ossification-annotated proteins

In our strategy, the IDRs from proteins with functional annotations related to the promotion of bone formation were extracted and used as input for a deep learning model to discover functional oligopeptides. The workflow is shown in Fig. 1a. The deep learning model comprised N-grams for mining the semantic pattern of natural functional oligopeptides and used Monte Carlo simulation to generate the new oligopeptides by extension (Fig. 1b). First, proteins were retrieved from UniProt based on their annotated involvement in bone formation using four osteogenic GO terms: “ossification”, “osteogenesis”, “osteoblast development”, and “osteoblast differentiation” (Fig. 1c). A total of 171 protein candidates were thus collected. (Table S1 and File 1) The IDRs of the 171 proteins were then identified using IUPred2A (File 2). The frequency distribution of AA residents between the full-length protein sequences and the IDRs showed a significant difference (Fig. 1d).

### Deep-learning-based identification of candidate osteogenesis-promoting oligopeptides

**Fig. 2:**
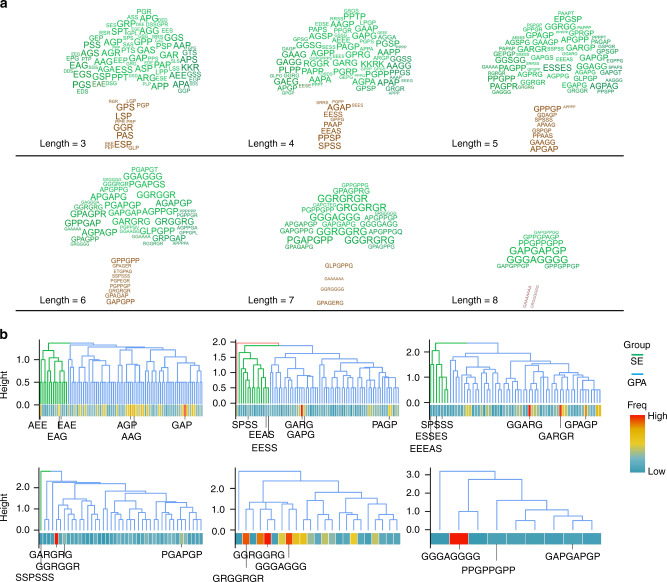
Functional oligopeptide candidates for promoting osteogenesis. **a** Illustration of the output results for osteogenic-related oligopeptides, shown as clustering trees. Oligopeptides (3–8 AA in length) were filtered according to their frequency in the IDRs and ranked by probability value inferred from the N-gram. The oligopeptide candidates, composed of the amino acids with a probability greater than random events (>1/20 for 20 types of human amino acids), were ranked by the product of conditional probabilities of each AA. The font size represents the rank of oligopeptides. **b** Oligopeptides of 3–8 amino acids in length clustered into two major groups: the SE subgroup and the GPA subgroup (the residents were mainly composed of S/E or G/P/A). The top-ranked oligopeptides in each subgroup at each length (a total of 28 oligopeptides) were selected for further experimental investigation of osteogenic activity

The N-grams mined semantic patterns among the IDRs and converted semantic pattern learnings into word context probability vectors. We then designed a Monte Carlo model to simulate the extension process from a single amino acid to oligopeptides of different lengths based on the probability vectors obtained from the N-gram analysis. Starting from the ten amino acids with the top-ranking frequency (Fig. 1d), oligopeptide candidates were obtained by extension (Table S2). To improve the efficiency of functional verification, we grouped the obtained oligopeptides by length and constructed a clustering tree for each group (Fig. 2a). These trees were divided into several subclusters (mainly composed of S/E or G/P/A), and we selected the top-ranked oligopeptides in each subcluster for functional verification (Fig. 2b).

### AIB5P promotes osteogenic differentiation of BMSCs in vitro and in vivo

**Fig. 3:**
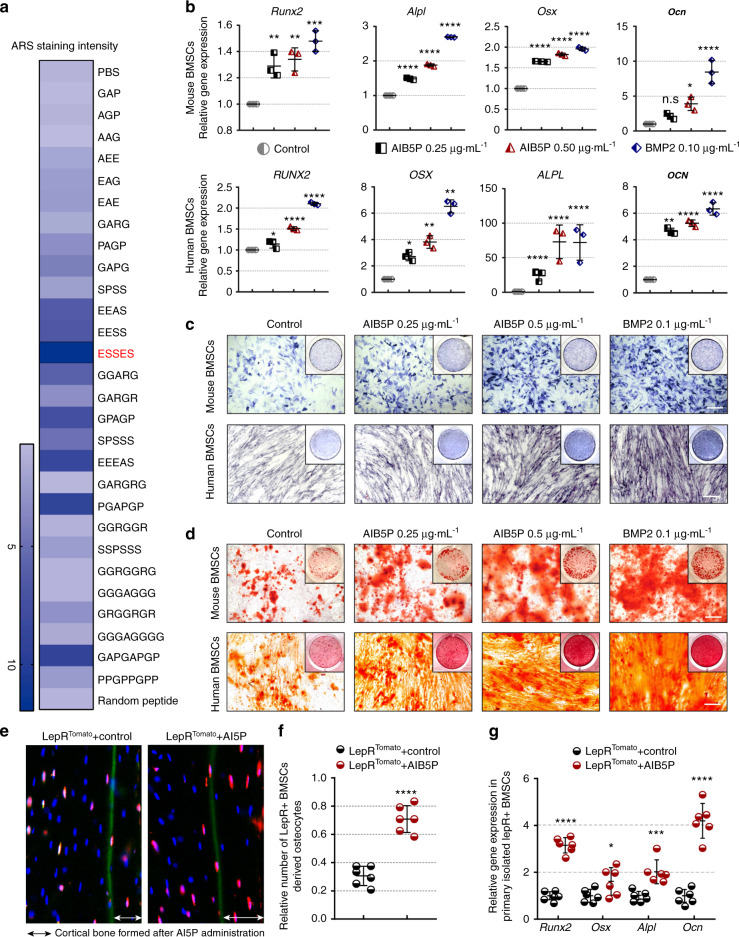
AIB5P promotes osteogenic differentiation of BMSCs. **a** Color assignment of ARS staining for oligopeptide candidate output by our deep learning model. **b** qPCR analysis of *Runx2*, *Alpl*, *Osx*, and *Ocn* expression levels in osteogenic-induced mBMSCs and hBMSCs after 1 week of AIB5P (0.25 μg·mL^−1^, 0.5 μg·mL^−1^) or BMP2 treatment (0.1 μg·mL^−^^1^). n = 3. **c** Alkaline phosphatase (ALP) staining of osteogenic-induced mouse BMSCs and human BMSCs after one week of AIB5P (0.25 μg·mL^−1^, 0.5 μg·mL^−1^) or BMP2 treatment (0.1 μg·mL^−1^). Bar, 100 μm. **d** Alizarin red (ARS) staining of osteogenic-induced mouse BMSCs and human BMSCs after 1 week of AIB5P (0.25 μg·mL^−1^, 0.5 μg·mL^−1^) or BMP2 treatment (0.1 μg·mL^−1^). Bar, 100 μm. **e** Cell lineage tracing of the LepR^+^ positive cells in newly formed cortical bone (representing LepR^+^-derived osteocytes). Calcein stained the bone deposition line when AIB5P administration started, and the double-headed arrow areas indicate red-positive osteocytes embedded within cortical bone. Bar, 100 μm. **f** The normalized number of deposited LepR^+^-derived osteocytes after 2 weeks of AIB5P treatment at a dose of 100 μg·kg^−1^ every 3 days. *n* = 6. The red cells in the calcein-labeled medial cortical bone are osteocytes derived from LepR^+^ cells. Bar, 25 μm. **g** *Runx2*, *Osx*, *Alpl*, and *Ocn* expression in LepR^+^ BMSCs sorted by flow cytometry. *n* = 6

We next selected an in vitro bone marrow mesenchymal stem cell (BMSC) osteoblastic differentiation model to evaluate the osteogenic activity of these developed oligopeptides. Alizarin red staining was used to test for osteogenesis-promoting activity. A majority of the output candidate oligopeptides obtained from the IDRs of osteogenic proteins could significantly accelerate BMSC osteogenic differentiation (Figs. 3a and S1A). We also compared the contribution of oligopeptides extracted from full-length protein sequences and IDRs to promoting osteogenesis (Fig. S1B and Table S3). The pentapeptide ESSES showed the strongest osteogenic activity among all 28 tested candidate oligopeptides. Based on its prominent function, we named this oligopeptide artificial intelligence-developed bone-forming pentapeptide (AIB5P).

We evaluated the osteogenic effects of AIB5P in both BMSCs and osteoblast osteogenic differentiation models. We found that AIB5P exposure could accelerate the osteogenic process in both cell types and that AIB5P showed much stronger effects on the osteoblastic differentiation of BMSCs than osteoblasts, as indicated by a much lower effective concentration of AIB5P on BMSCs than osteoblasts (Fig. S2). We then systemically evaluated the osteogenic activities in both mBMSCs and Human bone marrow mesenchymal stem cells (hBMSCs), and bone morphogenetic protein 2 was used as a positive control. As shown in Fig. 3b, AIB5P exposure significantly upregulated the expression of the osteogenic markers *Runx2*, *Osx*, *Alpl*, and *Ocn* in mBMSCs and hBMSCs. Consistently, ALP staining (Fig. 3c) and ARS staining (Fig. 3d) indicated that AIB5P treatment strongly enhanced the differentiation and mineralization of mBMSCs and hBMSCs. Previous studies have shown that BMSCs expressing the leptin receptor are a major source of stem cells contributing to bone formation in adult bone marrow.^11^ We, therefore, used LepR^td-tomato^ reporter mice to directly evaluate the osteogenic-promoting activity of AIB5P on BMSCs in vivo. At the beginning of the oligopeptide injection, all of the mice also received injection of calcein; this led to the deposition of a green fluorescent marker layer on the bone formation interface to indicate the starting point of treatment.^12^ Compared with the control treatment, 1.5 months of AIB5P treatment (100 μg·kg^−1^) significantly increased the number of newly formed osteocytes derived from LepR^+^ BMSCs on the inner surface of the calcein deposit (Fig. 3e, f). We also found that the expression of osteogenic markers in the LepR^+^ BMSCs sorted by flow cytometry indicated AIB5P-mediated promotion of osteoblast differentiation of BMSCs in vivo (Fig. 3g).

### AIB5P enhances bone formation in vivo

**Fig. 4:**
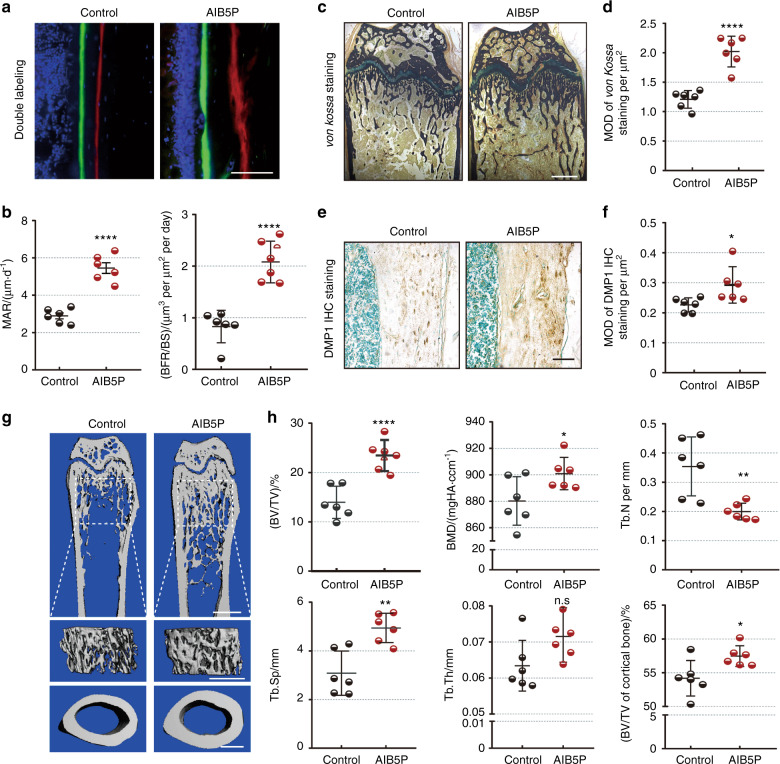
AIB5P enhances bone formation in vivo. Six mice in each group were administered AIB5P or vehicle (100 μg·kg^−1^, every 3 days, intravenous, total 1.5 months). Femurs were collected for further experiments. **a** High-magnification calcein and xylenol orange double labeling in femurs. Bar = 100 μm. The distance between the two fluorescent lines shows the amount of new bone deposition. **b** Quantification of the mineral apposition rate (MAR) and the bone formation rate per bone surface (BFR/BS) value. *n* = 6. **c, d** *von Kossa* staining and relative quantification of femur sections. Bar, 200 μm. Samples from six mice were used for statistics. **e, f** Representative anti-DMP1 IHC staining images and semiquantification. Samples from six mice were used for statistics. **g** Representative micro-CT of mouse femurs. Upper, longitudinal transverse sections at midshaft scans. Bar, 1 mm. Middle, trabecular bone underneath the growth plate. Bar, 500 μm. Lower, cortical bone. Bar, 500 μm. **h** Quantification analysis of micro-CT. Bone material density (BMD), bone volume per tissue volume (BV/TV), trabecular bone numbers (Tb.N), trabecular bone space (Tb.Sp) and trabecular bone thickness (Tb.Th) of trabecular bone compared between the vehicle control and AIB5P groups. BV/TV of the cortical bone was also compared. *n* = 6

We also conducted an experiment wherein wild-type mice were given intravenous injection of 100 μg·kg^−^^1^ AIB5P every 3 days. After 1.5 months of this treatment, the femurs of the AIB5P-treated mice were collected for dynamic histomorphometry measurements. Compared with that of the vehicle control group, the bone formation rate was significantly increased in the AIB5P treatment group (Fig. 4a, b). Consistently, the AIB5P-treated mice presented higher bone mass and mineralization levels, as revealed by *von Kossa* staining of the femurs (Fig. 4c, d). Anti-DMP1 immunohistochemical staining further indicated that the bone mineralization levels were obviously enhanced in the AIB5P-treated mice (Fig. 4e, f).

Next, micro-CT was performed to evaluate whether AIB5P-induced bone formation resulted in changes to bone mass. Indeed, AIB5P treatment significantly increased the trabecular and cortical bone mass of the mice (Fig. 4g). Consistently, quantitative analyses, including BMD, BV/TV, Tb.N, and Tb.Th, all showed significantly increased bone mass and density in the femurs of the AIB5P-treated mice (Fig. 4h). The bone mass of the fifth lumbar vertebrae (Fig. S3A) was significantly enhanced in the AIB5P group. Notably, TRAP staining showed that the increase in bone mass was not due to a decrease in either the number or surface area of osteoclasts (Fig. S4). These in vivo results collectively indicated that AIB5P treatment promotes bone formation and increases bone mass. Moreover, AIB5P showed no obvious toxicity effects in vivo, as demonstrated by analysis of serum biochemical markers, including ALS, ALT, BUN, and CK (Fig. S5B). There were no AIB5P treatment-related pathological changes in major organs, such as the heart, lung, liver, spleen, or kidney (Fig. S5C). We further analyzed the immune activity of oligopeptide injection and found that one day after AIB5P injection, the number and percentage of CD4^+^ T cells, CD8^+^ T cells, and B cells in the spleen were not significantly different from those in the control group (Fig. S5D). Intravenous injection of AIB5P had no significant effect on the body weight of mice. Taken together, these results demonstrate that AIB5P is an effective osteogenic oligopeptide that does not cause any obvious side effects.

### AIB5P increases bone mass in a bone loss model

**Fig. 5:**
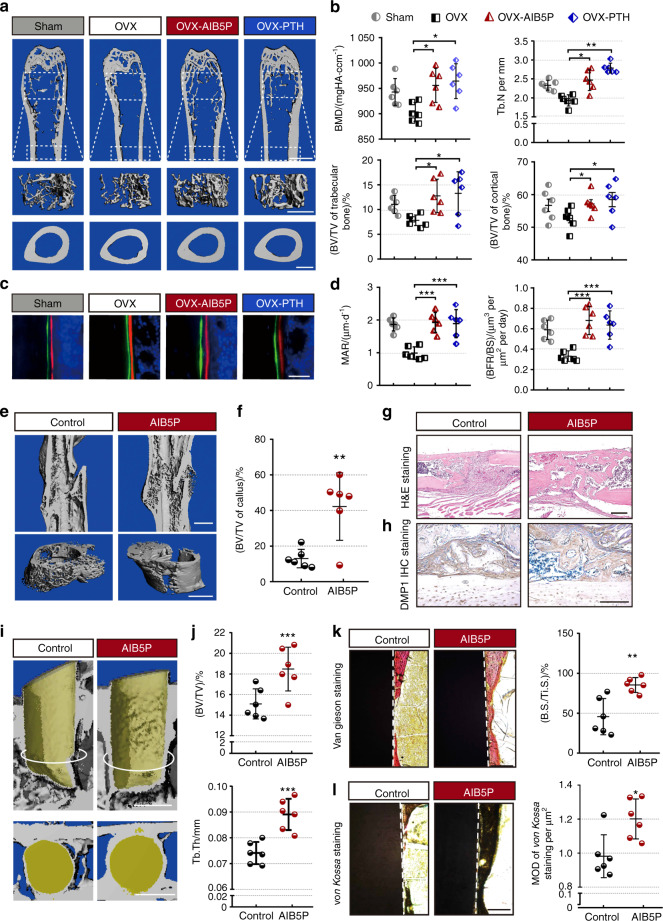
Evaluation of AIB5P treatment in multiple clinical bone loss/defect models. **a** Representative micro-CT of femurs from the OVX mice that received treatment with AIB5P (100 μg·kg^−1^, every 3 days, intravenous, total 1.5 months) or the known bone-formation-stimulating parathyroid hormone (PTH) (80 μg·kg^−1^, every 3 days, subcutaneous). Upper and middle panel, bar, 1 mm. Lower panel, bar, 500 μm. **b** Quantification of BMD, Tb.N, and BV/TV by micro-CT analysis. *n* = 6. **c, d** High-magnification images and quantification of calcein and xylenol orange double labeling of femur sections. Samples from six mice were used. **e** Upper, representative micro-CT images of femurs. Lower, representative micro-CT r statistics. Bar, 50 images of callus. Bar, 1 mm. **f** Quantification of BV/TV in callus. *n* = 6. **g, h** Representative H&E staining and anti-DMP1 IHC staining images of fractured femur sections. Bar, 200 μm. **i** Representative micro-CT images of vertical sections of implants (osteointegration model) treated with AIB5P (every 3 days, 100 μg·kg^−1^, intravenous, 3 weeks) or vehicle. The white circle line indicates the horizonal section position. Lower, cross-sectional view of bone formation surrounding the implant. Bar, 1 mm. **j** Quantification of new bone thickness on the implant surface. *n* = 6. **k** Van Gieson staining and bone quantification of the midshaft surface of implants. Bone surface per titanium implant surface (B.S./Ti.S.). Bar, 100 μm. *n* = 6. **l** *von Kossa* staining images and relative quantification of new bone formation in the implant model. Bar, 100 μm. *n* = 6

We investigated the effect(s) of AIB5P in an ovariectomy-induced osteoporosis (OVX) model.^13^ We used human parathyroid hormone (PTH), which is known to stimulate bone formation (1–34), as a positive control in this model (80 μg·kg^−1^, every three days, subcutaneous). Femurs were harvested from the OVX mice that had received 1.5 months of AIB5P treatment (100 μg·kg^−1^, every 3 days, intravenous). Micro-CT scanning showed that the trabecular bone mineral density, cortical bone mineral density, and bone volume of the AIB5P treatment group were all significantly increased compared to those of the vehicle control-treated OVX model animals (Fig. 5a, b). Both bone masses of the fifth lumbar vertebrae (Fig. S3B) and the bone formation rate were significantly enhanced in the AIB5P group (Fig. 5c, d). The therapeutic effects of AIB5P on the OVX model were similar to those conferred by PTH (Figs. 5a–d, S3A, B).

### AIB5P accelerates bone healing in a fracture model

We next tested whether the osteogenesis-promoting effects of AIB5P facilitate fracture bone repair. Briefly, one week after fracture, AIB5P was injected intravenously into mice for two weeks; micro-CT scanning was performed at the end of the third week. The data showed denser calli at the fractured position in the AIB5P-treated mice than in the vehicle control mice (Fig. 5e, f). Consistently, H&E and anti-DMP1 IHC staining revealed increased coalesced cortical bone thickness and higher mineralization levels in the fractured area of the AIB5P group (Fig. 5g, h).

### AIB5P promotes osteointegration in a titanium implant model

We also established a mouse model of titanium implantation to evaluate the effect(s) of AIB5P on implant osseointegration in mice. We found that three weeks of AIB5P treatment significantly promoted the osseointegration and surface bone thickness of titanium implants compared to those of the vehicle control-treated animals (Fig. 5i). The BV/TV and Tb.Th metrics of the bone around the implants were substantially increased in the AIB5P group (Fig. 5j). Both *von Kossa* staining (Fig. 5k) and Van Gieson staining (Fig. 5l) showed that the bone mass and mineralization of the newly formed bone covering the implant surface were obviously increased in the AIB5P-treated mice compared to the controls. Together, these results indicate that AIB5P could be used as a “therapeutic oligopeptide” for the treatment of multiple bone diseases.

### AIB5P interacts with integrin α5 to promote osteogenesis

**Fig. 6:**
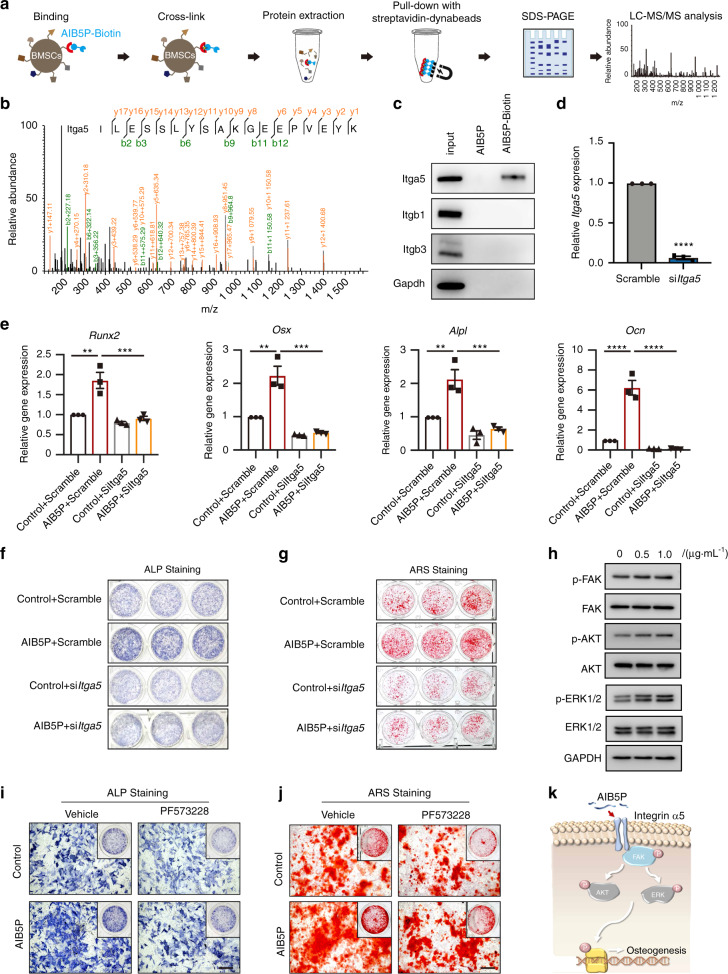
AIB5P interacts with integrin α5 to promote osteogenesis. **a** Schematic diagram of the biotinylated peptide pulldown/MS assay. **b** Mass spectrometry analysis identified Itga5, which was pulled down from BMSC lysates by biotin-labeled AIB5P. **c** Representative western blots of Itga5 pulled down by biotin-labeled AIB5P. **d** qPCR analysis of siRNA-mediated *Itga5* gene silencing efficiency. *n* = 3 **e** qPCR analysis of known osteogenic markers in AIB5P-treated, *Itga5* knockdown BMSCs. *n* = 3 **f** ALP staining and **g** ARS staining of AIB5P-treated, *Itga5* knockdown BMSCs. The experiment was repeated three times independently with similar results. **h** Representative western blots of FAK signaling component proteins in AIB5P-treated BMSCs. The experiment was repeated three times independently with similar results. **i** ALP staining and **j** ARS staining of AIB5P-treated BMSCs in the presence of the Itga5 inhibitor PF573228 (10 μmol·L^−1^). The experiment was repeated three times independently with similar results. Bar, 100 μm. **k** Schematic diagram of AIB5P-related osteogenic pathways

Finally, to explore the mechanism(s) through which AIB5P promotes osteogenesis, we analyzed the subcellular distribution of AIB5P using immunofluorescence. Interestingly, we found that FITC-labeled AIB5P was distributed on the cell surface of BMSCs (Fig. S6). This finding indicates that AIB5P may function as a peptide ligand of some cell surface receptor(s). Pursuing this hypothesis, we performed a biotinylated peptide pulldown/MS assay to help identify the target protein(s) that may interact with AIB5P (Fig. 6a). Integrin α5 (Itga5) was identified as an interaction target of AIB5P in BMSCs (Fig. 6b). The coverage of the identified peptides reached 28.1% of Itga5. We also verified the specific interaction between AIB5P and Itga5 with immunoblotting: AIB5P interacted with Itga5 but not with other examined integrin proteins (Fig. 6c).

We next used siRNAs to knock down *Itga5* expression in BMSCs to verify whether AIB5P osteogenic activity is mediated by Itga5 (Fig. 6d). *Itga5* knockdown was successful, and we found that the knockdown cells had a reduced extent of AIB5P-mediated osteogenic marker gene induction compared to that of the AIB5P-treated WT cells (Fig. 6e). Consistently, both ALP staining (Fig. 6f) and ARS staining (Fig. 6g) also indicated that *Itga5* knockdown blocked the osteogenic activities of AIB5P in BMSCs. These results identify Itga5 as a functional receptor for AIB5P in BMSCs.

Next, we analyzed the downstream pathways stimulated by the AIB5P-Itga5 interaction and noted that the phosphorylation-mediated activation of FAK and the FAK downstream kinases AKT and ERK1/2 was increased upon AIB5P treatment (Fig. 6h). Finally, we used chemical inhibition of FAK to confirm the functional involvement of FAK activation in the observed AIB5P-induced osteoblast differentiation. After pretreatment with the FAK inhibitor PF-573228,^14^ AIB5P-induced ALP activity and calcium deposition were substantially decreased (Fig. 6i, j). Collectively, these results reveal that the osteogenic effects of AIB5P result from its reception by Itga5 and the attendant activation of FAK signaling (Fig. 6k).

## Discussion

The lack of sufficient datasets and informative features are two major problems hindering AI-assisted oligopeptide drug development. As the probable solution to a lack of sufficient datasets will require long-term data acquisition efforts by the pharmaceutical industry, at this stage, it is logical to focus on identifying effective features. AlphaFold2 has very recently drawn extensive public attention,^15^ and owing to its strong performance in predicting protein structures, this powerful tool will soon be used in both peptide and protein research, including peptide drug R&D. However, notably, owing to the lack of rigid and nonvariable three-dimensional structures for oligopeptides, the currently available structural models of peptides are of limited utility for the design of oligopeptide drugs. Additionally, the short sequences of oligopeptides make it difficult to design distinguishable manual features. Therefore, end-to-end models where effective features can be learned in small sample cases represent one of the few apparently tenable options.

NLP algorithms have progressed rapidly with the rise of deep learning, from the classic N-gram^16^ and RNN^17^ to the more advanced Transformer^18^ and BERT.^19^ The more advanced models have better long-distance semantic pattern mining ability, but these also require more training data. Given the lack of available data and long semantic patterns, we used a basic N-gram model for oligopeptides to mine semantic patterns based on IDRs. An N-gram model is essentially a conditional probability calculation model that is somewhat similar to naive Bayes analysis, but it calculates probability via a deep neural network. The model is simple in principle, with no need for large training data, but is highly efficient in inference performance, helping explain its wide use in NLP tasks to date.^20^ Supporting the utility of an N-gram model for oligopeptide drug discovery, we obtained multiple oligopeptides with obvious bone formation-promoting bioactivity.

Although the dictum that “form follows function” is a central tenet of structural biology, it is now quite clear that the conformational malleability of IDRs enables them to perform very specialized functions that cannot be accomplished by globular proteins.^9,21,22^ Generally, IDRs exert the following functions as part of the cellular signaling machinery: (1) recognize proteins and nucleic acids, accelerate chemical reactions between bound partners, and promote the accommodation of post-translational modifications, alternative splicing, protein fusions, and insertions or deletions^23^; (2) add complexity to regulatory networks^24^; and (3) facilitate phase transition and heterochromatin functions in cells.^25^ A common functional module within IDRs is the so-called “linear motif”, which is usually 3–10 amino acids long.^26^ The length of an IDR linear motif is notably similar to that of an oligopeptide. We utilized our artificial intelligence model to screen oligopeptides in both IDRs and non-IDRs, and both regions recommended oligopeptide candidates (length from three amino acids to ten amino acids). Our model recommended more oligopeptides in IDRs than in non-IDRs (361 vs. 244). Our use of an N-gram model and subsequent analyses resulted in the identification of more than twenty oligopeptides between 3 and 10 aa in length (developed from IDR sequences of osteogenic proteins) that are effective in promoting the differentiation of bone marrow stem cells. Functional verification of the recommended peptides showed that the peptides in the IDR showed better osteogenic effects on BMSCs (Fig. S1). The most effective of these compounds (AIB5P) was selected for subsequent experiments, which showed their impressive osteogenic effects both in vivo and in vitro.

Integrin family proteins are important regulators of migration, adhesion, survival, and differentiation of BMSCs.^27–29^ Our mechanistic analysis indicated that the AIB5P-integrin FAK signal mediates the differentiation of BMSCs. Integrin α5 is highly expressed in BMSCs and is closely involved in the bone formation process.^30,31^ Silencing of the expression of integrin α5 abrogated the osteoblast differentiation of BMSCs, whereas its overexpression by lentivirus markedly promoted osteogenesis in vivo.^32,33^ Notably, stimulating integrin α5 could induce osteogenesis of BMSCs by peptide ligand,^34^ monoclonal antibody SNAKA51^32^ or chemical compounds.^35^ In this study, we found that the oligopeptide AIB5P could regulate the osteogenesis of BMSCs by directly binding to integrin α5. Silencing integrin α5 and inhibiting its downstream kinase FAK completely inhibited the osteogenic activity of oligopeptides, indicating that the AIB5P-integrin α5-FAK signal mediates the differentiation and osteogenesis of BMSCs. Thus, our findings further highlight that stimulating integrin α5 is a promising strategy for oligopeptide-induced osteoblast differentiation and bone formation.

This study systematically explored the osteogenic activity of AIB5P in vivo and in vitro, but there are still some shortcomings, such as the detection of the lowest effective dose of AIB5P and the further modification of oligopeptides to reduce the injection frequency. Currently, some strategies have been proven to be effective in prolonging the serum residence time of peptide drugs, such as peptide acylation (as seen in the GLP-1 agonist Victoza), insertion of albumin-binding peptide elements in the peptide backbone, conjugation to albumin-binding antibody fragments (AlbudAbTM technology) and polyethylene glycol (PEG)-ylation.^36^ Since AIB5P contains a small number of amino acids, changes in the function of oligopeptides can be easily generated by amino acid substitutions. We are currently using amidation, PEG modification, and cyclized peptides to prolong the serum residence time of AIB5P peptides.

In summary, we have developed a natural language processing N-gram model to learn functional oligopeptides from the IDRs of proteins with functional annotations related to bone formation. It should be straightforward to substitute proteins with functional annotations for any biological process of interest for the bone-formation-related set of proteins we examined in this study. The diverse functions of IDRs indicate that focusing discovery efforts on these sequences can be an effective form of input to yield functional oligopeptides. Thus, we envision that our DL-based discovery strategy can substantially accelerate the development of oligopeptide drugs for the treatment of many clinical indications in the near future.

## Materials and methods

### A subset of proteins promoting bone formation

To obtain proteins characterized by bone formation, we downloaded all proteins listed in the GO terms of ossification, osteogenesis, osteoblast development, osteoblast differentiation, and osteoblast proliferation from UniProt (https://www.prot.org/downloads). We retained 171 of them by further confirming their ability to promote bone formation (Table S1).

### IDR prediction

We submitted all protein sequences to *IUPred2A*^37^ to identify intrinsically disordered protein regions (IDRs) under the module of “long disorder” with the parameter “context-dependent predictions (default ANCHOR2)”. We kept those continuous sequences with IUPred scores larger than 0.5 as the IDR for each protein. We also divided the IDR into two parts based on the presence or absence of disordered binding regions, which were supported by the ANCHOR score. However, no improvement was detected for functional peptide exploration (data not shown) with or without disordered binding regions.

### Functional peptide learning using a deep neural network

#### N-gram model

To obtain the potential functional peptide, we employed a deep learning method based on the N-gram model. First, we trained the model by taking the word vectors (under the current circumstance of a protein sequence, word vectors refer to amino acid vectors) of context as inputs. We then used the trained model to generate the probability distribution of the next word and thus referred to the corresponding conditional probability. More specifically, the N-Gram model computes each conditional probability (*p*) by using the formula below:

$$p(\omega _k|context(\omega _k)) = F\left( {i_{\omega _k},v\left( {context(\omega _k)} \right),\theta } \right)$$

where *F* indicates the deep neural network, *θ* those parameters to be optimized in *F*, $i_{\omega _k}$ the serial number of the *k*-th word *ω*_*k*_ in the set of amino acids, and *v*(context(*ω*_*k*_)) the word vector presentation of the context(*ω*_*k*_) of *ω*_*k*_. Each layer of the model is described as follows:

#### Input layer

In this layer, every word was mapped into a word vector with a length of *m*. Notably, the word vectors were initialized randomly before training and were iterated during the training process.

#### Projection layer

As input of the hidden layer, all word vectors were joined into a long vector in this layer. For instance, if $context(\omega _k) = \left\{ {\omega _{k - n + 1}, \cdots ,\omega _{k - 1}} \right\}$, then $v\left( {context(\omega _k)} \right) = \left[ {v\left( {\omega _{k - n + 1}} \right), \cdots ,v\left( {\omega _{k - 1}} \right)} \right]$ where the vector dimension of context with *n*–1 words is *m*(*n*–1). Additionally, context with words less than *n*–1 can be padded as the normal vector.

#### Hidden layer

For extraction of the deep features, outputs of the projection layer were delivered into such a layer. We designed a hidden layer with a size of 128 and employed a tanh function for activation.

#### Output layer

Designed for distributary, this layer changed the output of the hidden layer into a vector with a size of *N*, where *N* is the number of possible results we set.

#### Softmax layer

With the results of the output layer as input, this layer normalized the results and output a new vector, of which each item was the probability of the corresponding results.

#### Monte Carlo Model

As mentioned above, based on the maximum probability value in the probability vector of the softmax layer, the N-gram model refers to the most likely result. However, there are some drawbacks to this inference process. For instance, if we obtain a vector with a maximum value of 0.4 and a second value of 0.39, the N-gram model will recommend a result corresponding to 0.4 as the output. However, the result corresponding to 0.39 with almost equal possibility is omitted. Furthermore, such an inference process is similar to a greed-based method, with a so-called optimized result as the output for each call. Thus, the combination of optimized results for each call never means a global optimum. In addition, such a process can obtain only one result but more possible results to be chosen according to our needs.

Considering these factors, we introduced the Monte Carlo simulation method to obtain more natural results. Instead of taking the maximum value as a result, the Monte Carlo method takes the probability vector of the softmax layer as input and then generates many results by throwing the dice, of which each face presents a potential result with the corresponding probability in the probability vector.

Taking one word as a start, we first called the N-gram model to obtain the probability vector and then used Monte Carlo simulation to generate the next protentional words. Splicing the input word and these protentional words as a new word for the next input, we repeated the process above until the length of eventual output. Finally, all these simulation results were sorted by their products of conditional probability in each cycle, and the top 100 oligopeptide candidates, composed of the amino acids with probability larger than random events (> 1/20 for 20 types of human amino acids), were sent for cluster tree construction.

### Hierarchical clustering based on Levenshtein distance

To further excavate functional oligopeptides from deep-learning-based oligopeptides, we applied hierarchical clustering based on the Levenshtein distance between any two equal-length oligopeptides. The main reasons included the following: 1) homogenous sequences are more likely to obtain similar scores in the NLP model, which makes certain homogenous sequences tested multiple times; 2) clustering among sequences of different lengths may introduce large deviations, as the missing parts in the short ones will be treated as arbitrary amino acids; and 3) clustering has the ability to discover new information. Briefly, we first used the “adist” function to calculate the Levenshtein distance between any two oligopeptides of the same length. Second, we clustered these oligopeptides using the “hclust” function. Third, we plotted the dendrogram using the phylogram and ggtree packages. Finally, based on the results of hierarchical clustering, we chose the top three oligopeptides in every group as candidates in the subsequent experimental validations. All the analyses were integrated into a homemade R script.

### Synthesis of oligopeptide

Peptides with purity >99% were ordered from China Peptides Co., Ltd., each 500 mg. HPLC and MALDI data were provided with lyophilized peptides. Peptides used in the in vitro assay were dissolved in saline to a final concentration of 0.1 mg·mL^−1^ stock solutions and stored at –80 °C for no more than two weeks. The random sequence AGLAS peptide and human BMP-2 (Accession # NP_001191) were synthesized as controls for in vivo assays.

### Cell culture and treatments

Mouse primary BMSCs were isolated from 8- to 12-week-old C57BL/6 mice following previously reported protocols.^38^ hBMSCs (American Type Culture Collection ATCC) were obtained from ATCC. BMSCs were cultured in 5% CO_2_ at 37 °C in α-MEM (Gibco) supplemented with 10% FBS (Gibco, Carlsbad, CA) and 1% penicillin and streptomycin (Life Technologies). For determination of the osteogenic activities of the peptides, mouse BMSCs, osteoblasts, and MC3T3-E1 cells were cultured in cell culture plates overnight at a density of 2 × 10^5^ cells per mL and then treated with the indicated concentrations of peptides in osteogenic induction medium for different days. The osteogenic induction medium was renewed every other day.

### Animals

All animal studies were carried out in accordance with the guidelines of the Institutional Animal Care and Use Committees of Tongji University. Hauschka Ha/ICR female mice were used in this study as wild-type mice to generate osteoporosis, bone fracture and implant osteointegration models. Other mice used in this study included LepR^td-tomato^ mice (*LepR*-Cre mice^38^ cross *loxp-Tomato* mice^39^).

### ALP and ARS staining

ALP staining was assayed using a BCIP/NBT Alkaline Phosphatase Color Development Kit (Beyotime) according to the manufacturer’s instructions. Briefly, BMSCs that differentiated for 7 days were fixed with 4% paraformaldehyde (PFA) for 10 min. Then, the cells were washed and soaked in ALP staining buffer for 10 min at RT. For ARS staining, BMSCs that differentiated for 21 days were washed with PBS two times and fixed with 4% PFA for 10 min at RT. Then, the cells were soaked in ARS staining buffer for 10 min at RT. All images of the plates were acquired by an Olympus microscope, and quantitative data were collected through *ImageJ*.

### Administration of AIB5P to wild-type/LepR^td-tomato^ mice

Briefly, AIB5P was intravenously injected in 12-week-old Hauschka Ha/ICR female mice or LepR^td-tomato^ female mice at a dose of 100 μg·kg^−1^ every three days. The mice were sacrificed after 1.5 months of treatment.

### Micro-CT analysis

The distal femurs of mice from each group were scanned ex vivo using a micro-CT system (micro-CT50, Scanco Medical, Switzerland). For visualization of all bone mass changes in the distal femur, the total femur at a voxel size of 14 μm was scanned and reconstructed. Furthermore, 100 slices of trabecular bone underneath the growth plate (1.4 mm), 50 slices of vertebral body (0.7 mm) and 50 slices of the cortex bone area (0.7 mm) at a voxel size of 14 μm were reconstructed for the statistical analysis. For callus analysis of fracture models, the callus of 25 slices above and below the fracture line was selected as the region of interest. In the micro-CT slice scan interface, the bone tissue outside the cortical bone of each slice near the fracture line was judged to be callus. Sigma = 1.2, supports = 2 and threshold = 200 were used to calculate the following parameters: BV/TV, Tb.Th, Tb.Sp Tb.N, and BMD. The fifth lumbar vertebrae in the control and WT mice were compared by micro-CT analysis.

### Double-labeling analysis

We investigated the impact of AIB5P on the bone formation rate. Xylenol orange and calcein labeling was performed. Briefly, first, 10 mg·kg^−1^ xylenol orange was injected into the mice via intraperitoneal injection, and calcein was injected after 2 weeks at a dose of 10 mg·kg^−1^.^12^ Then, 12 h after the injection of calcein, all mice were sacrificed. The femurs were collected and fixed in 4% PFA solution for 24 h. Then, the samples were embedded in light-cured resin (EXAKT 7200 VLC, Germany) for 13.5 h before hard tissue sectioning was performed with a hard tissue sectioning and grinding system (EXAKT micro section and grinding system, Germany). The thicknesses of all slides were controlled between 15 μm and 20 μm. The slides were rinsed and counterstained with DAPI (Sigma, USA). Images were obtained under a confocal microscope (Leica image analysis system, Q500MC). Bone dynamic histomorphometric analyses for MAR and BFR/BS were performed according to the standardized procedures published by the American Society for Bone Mineral Research.^40^

### Hematoxylin and eosin staining

The femurs from the mice of the control or AIB5P-treated groups were fixed with 4% PFA solution, decalcified in 10% EDTA for 21 days, embedded in paraffin and sectioned at a thickness of 4 µm. Finally, hematoxylin and eosin (H&E) staining was performed according to the manufacturer’s protocol.

### *von Kossa* staining

Hard tissue sectioning was performed on distal femurs from the mice of the control or AIB5P-treated groups embedded in light-cured resin. The femurs were collected and fixed in 4% PFA solution for 24 h. Then, the samples were embedded in light-cured resin (EXAKT 7200 VLC, Germany) for 13.5 h before hard tissue sectioning was performed with a hard tissue sectioning and grinding system (EXAKT micro section and grinding system, Germany). The thicknesses of all slides were controlled between 15 μm and 20 μm. The slides were exposed to strong light until the mineralized bone turned black after applying 2% silver nitrate solution. Then, the slides were rinsed with distilled water and quickly dipped into 5% sodium thiosulfate. The sections were viewed under a light microscope, and the mineral apposition rate was determined by bone dynamic histomorphometric analysis.

### Immunohistochemistry

Bilateral femurs were dissected, fixed with 4% PFA, and embedded in paraffin. For detection of DMP1 in the matrix, the slides were incubated with an anti-DMP1 antibody (monoclonal, 8G10.3) diluted 1:300 in goat serum overnight at 4 °C, followed by rinsing and incubating with a rabbit anti-mouse secondary antibody (MXB, KIT-9706, China) for 30 min. The immunoreactivity to antibodies was visualized using a DAB kit following the manufacturer’s instructions. The sections were counterstained with methyl green and viewed under a light microscope.

### Toxicity assays

To detect the toxicity of AIB5P to important viscera, we treated wild-type mice with 10 mg·kg^−1^ control peptide or AIB5P at a frequency of once every three days for one and a half months. Serum, heart, lung, liver, spleen, and kidney were collected after administration. The organs were sliced after embedding in paraffin. H&E staining was performed for histopathological analysis. Serum was used for ELISA detection to detect the expression of biochemical factors.

### Van Gieson staining

The sections from the implant model were fixed with 4% PFA solution and then decalcified in 10% EDTA for 21 days. Paraffin embedding and sectioning at a thickness of 4 µm were performed. Finally, Van Gieson staining was performed according to the manufacturer’s protocol. Briefly, the dewaxed slices were stained in hematoxylin for ~3 min and then washed with water for 10 min. After dying with Van Gieson staining solution for 2 min, the sections were rapidly differentiated for several seconds with 95% ethyl alcohol’s semen. Then, the sections were dehydrated in absolute ethyl alcohol, made transparent in xylene, and sealed. Finally, imaging was performed.

### Quantitative real-time PCR (q-PCR) assay

The total RNA was isolated from tissues or cells with RNAiso Plus reagent (TaKaRa). cDNA was synthesized using a PrimeScript™ RT reagent Kit with gDNA Eraser (Perfect Real Time) (TaKaRa). q-PCR was conducted with a SYBR Premix Ex Taq II kit (TaKaRa). The levels of mRNAs were normalized to that of the housekeeping gene *Gapdh*. All q-PCR procedures, including the design of the primers, validation of PCR conditions and quantification, were performed according to MIQE guidelines. Gene-specific primer sequences are listed in Supplementary Table 4.

### Therapeutic evaluation of AIB5P in a mouse model of osteoporosis

For the bone loss rescue analysis, 12-week-old female ICR mice were ovariectomized. After 3 months, all OVX mice were divided into the following four groups (six mice per group): the sham, OVX, OVX + PTH, and OVX + AIB5P groups. In the OVX or OVX + AIB5P group, the OVX mice were treated with a control peptide and AIB5P at a dose of 100 μg·kg^−1^ every three days. For the OVX + PTH group, the OVX mice were intraperitoneally injected with PTH at a dose of 80 μg·kg^−1^ every 3 days. First, 75% ethanol was used to prepare the injection site. Then, we placed an aseptic needle under the abdomen, right or left quadrant at an angle of 30°. The needle was aspirated to ensure that it was placed correctly, and the solution was slowly injected. One month after drug administration, all mice were sacrificed.

### Bone fracture model

An established fracture model was generated in 12-week-old female ICR mice. Briefly, after anesthesia with isoflurane inhalation, a surgical blade was used to transect the middle diaphysis of the femur. In addition, the fracture site was stabilized by inserting a 0.7-mm sterile needle. The periosteum adjacent to the fracture site was carefully protected to avoid human interference. The fractured mice were injected with AIB5P at a dose of 100 μg·kg^−1^ every three days from the first weekend after surgery. The mice were sacrificed 3 weeks post-fracture, and the femur fracture specimens were fixed in 4% paraformaldehyde overnight at 4 °C.

### Implant osteointegration model

A titanium implant osteointegration model in the femurs of 12-week-old female ICR mice was established to evaluate the therapeutic potential of AIB5P for promoting osseointegration. Briefly, after anesthesia with isoflurane inhalation, a 1.5 cm incision was made directly above the femur. The fascia and muscle were separated, and the middle part of the thigh was exposed to drill a 1.5 mm loophole with a low-speed handset. The muscles and skin were sutured after the titanium nail, which was 1.5 mm in diameter and length of 3 mm, was inserted into the notch. AIB5P administration was performed from week 2 to week 4 after the surgery at a dose of 100 μg·kg^−1^ every 3 days.

### Western blot assay

The protein concentration was quantified by the BCA Protein Assay Kit (Beyotime) and normalized. Approximately 30 µg of protein extracts was analyzed by electrophoresis using a 12% SDS–PAGE gel and electroblotted onto polyvinylidene fluoride membranes (Millipore). The membranes were blocked with 5% BSA and incubated with specific primary antibodies overnight at 4 °C with gentle rotation. A horseradish peroxidase-labeled secondary antibody was added, incubated for 1 h with shaking and visualized using an enhanced chemiluminescence reagent (Millipore). The primary antibodies included anti-GAPDH (#200306-TE4, 1:2 000), anti-FAK (#860324, 1:1 000), anti-p-FAK (#381143, 1:1 000), anti-ITGB1 (#R24729, 1:1 000), anti-ITGA5 (#R24725, 1:1 000), and anti-ITGB3 (#384730, 1:1 000) from Zen BioScience and anti-AKT (#4691, 1:1 000), anti-p-AKT (#4060, 1:1 000), anti-ERK (#4695, 1:1 000), and anti-p-ERK (#4370, 1:1 000) from Cell Signaling.

### Biotinylated peptide pulldown assay

Biotinylated peptide pulldown assays were performed following previously reported protocols with brief modifications.^41^ Briefly, BMSCs were cultured in 100 mm cell culture dishes to 90% confluence. Then, the cells were washed once with ice-cold PBS, and 500 μg C-terminal biotinylated AIB5P was added to the dishes and incubated for 1 h at 4 °C. The cells were then incubated with 2 mmol·L^−1^ DTSSP solution on ice for 2 h to perform crosslinking. Next, the reaction was stopped by the addition of 20 mmol·L^−1^ Tris-HCl. Then, the total proteins were extracted by 1 mL of IP lysis buffer (Thermo) on ice for 10 min. Dynabeads MyOne Streptavidin C1 (Invitrogen) was added to the protein lysates and mixed thoroughly. After incubation at 4 °C overnight with gentle rotation, magnetic separation was used to pull down the peptide-protein complexes. After the complex was washed four times with lysis buffer, the purified proteins were separated by SDS-PAGE, the protein band was digested with trypsin, and the peptide fragments were identified by LC/MS/MS (Q Exactive, Thermo Scientific).

### Immunofluorescence assay

BMSCs were incubated with 10 μg·mL^−1^ FITC-tagged AIB5P for 2 h, and then, Hoechst 33342 (Beyotime) was added to the plate to label the nuclei. Finally, the cells were rinsed two times with ice-cold PBS and visualized under a confocal laser scanning microscope (Zeiss, LSM700).

### Cell transfection

JetPRIME transfection reagent (Polyplus) was used for siRNA transfection. Briefly, 110 pmol siRNA was added to 200 μL of jetPRIME buffer and vortexed for 10 s. Then, 4 μL of jetPRIME transfection reagent was added to the buffer, vortexed for 1 s, spun down and incubated for 10 min at RT. Finally, the transfection complex was added to the 6-well plate and incubated for 6 h. Then, the medium was changed, and the cells were cultured for 48 h before sampling. Scramble (TTCTCCGAA CGTGTCACGT) and Itga5 siRNAs (CACUGUUCCUCAUCUUCAAGA) were synthesized by IBSBio (Shanghai, China).

### Statistics

All statistical analyses were performed with SPSS v16.0. Significant differences between two groups were determined by unpaired Student’s *t* test (two-tailed). Significant differences among multiple groups were determined by one-way ANOVA with Dunnett’s multiple comparisons test. All numerical data are expressed as the mean ± s.e.m. *P* < 0.05 was considered statistically significant. *P < 0.05, **P < 0.01, ***P < 0.001, ****P < 0.000 1, n.s., not significant.

### Study approval

This study was approved by the Ethics Committee and the Institutional Animal Care and Use Committee of Tongji University (2019-TJ2019015). All animals were bred according to the National Institutes of Health’s Guide for Care and Use of Laboratory Animals.

## Supplementary information

Table S1

Table S2

Table S3

Table S4

Figure S1

Figure S2

Figure S3

Figure S4

Figure S5

Figure S6

## Data Availability

Reasonable requests for additional data or materials will be fulfilled under appropriate agreements.
