# Supplementary material for: Generation of functional oligopeptides that promote osteogenesis based on unsupervised deep learning of protein IDRs
Source: Bone Res. 2022 Mar 1;10:23. doi: 10.1038/s41413-022-00193-1 (PMC8885677; doi:10.1038/s41413-022-00193-1)
Supplement: Supplementary file 4 — Table S4 [file 41413_2022_193_MOESM4_ESM.docx]

**Table S4. Primers used for Q-PCR.**

| **Primer name** | **Primer sequence** |
| --- | --- |
| Mouse Gapdh F | AGGTCGGTGTGAACGGATTTG |
| Mouse Gapdh R | TGTAGACCATGTAGTTGAGGTCA |
| Mouse Runx2 F | TTCAACGATCTGAGATTTGTGGG |
| Mouse Runx2 R | GGATGAGGAATGCGCCCTA |
| Mouse Alpl F | GAGCGTCATCCCAGTGGAG |
| Mouse Alpl R | TAGCGGTTACTGTAGACACCC |
| Mouse Ocn F | TTTCTGCTCACTCTGCTGACC |
| Mouse Ocn R | GCCGGAGTCTGTTCACTACC |
| Mouse Osx F | GGCGTCCTCTCTGCTTGAG |
| Mouse Osx R | TTCCCCAGGGTTGTTGAGTC |
| Mouse Itga5 F | AAAATCTGGGTGAGGGCGGTG |
| Mouse Itga5 R | CAGAGACTGGTGCCTGCCTTC |
