# Supplementary figures and images for: Generation of functional oligopeptides that promote osteogenesis based on unsupervised deep learning of protein IDRs
Source: Bone Res. 2022 Mar 1;10:23. doi: 10.1038/s41413-022-00193-1 (PMC8885677; doi:10.1038/s41413-022-00193-1)

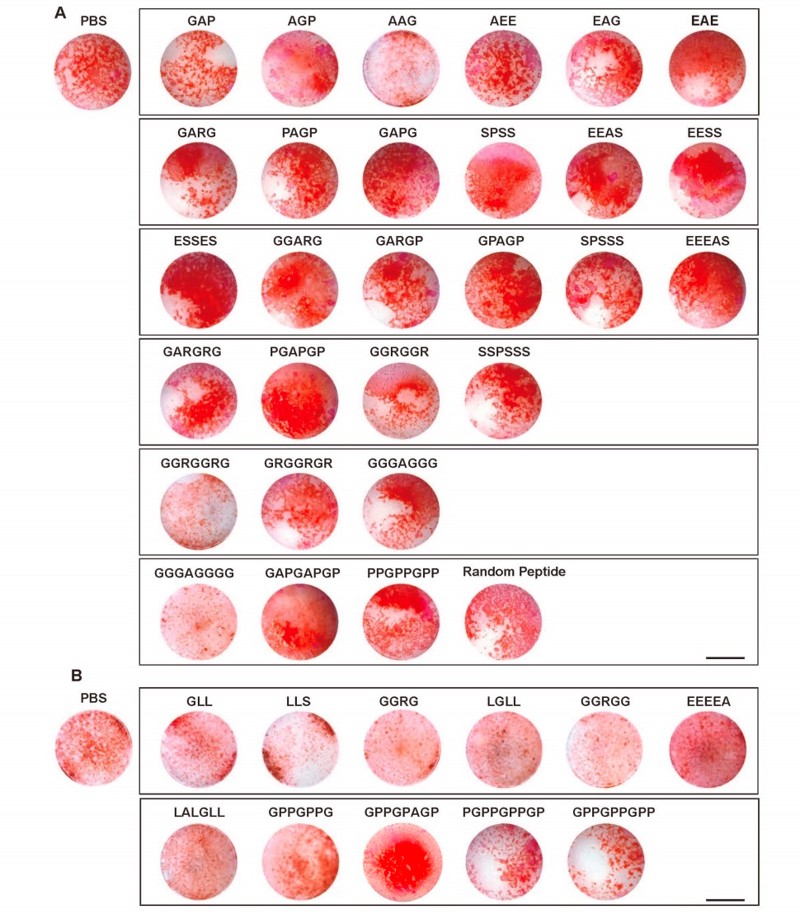

Supplement: Supplementary file 5 — Figure S1 [file 41413_2022_193_MOESM5_ESM.jpg]

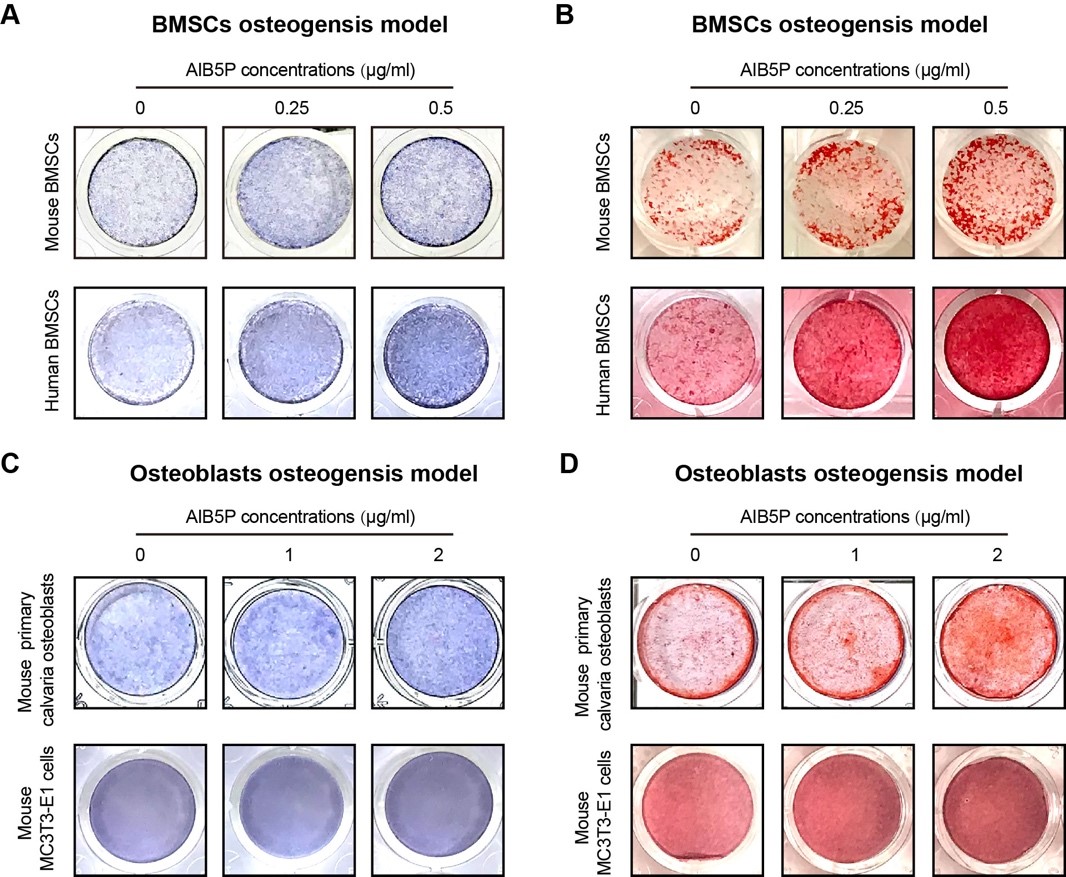

Supplement: Supplementary file 6 — Figure S2 [file 41413_2022_193_MOESM6_ESM.jpg]

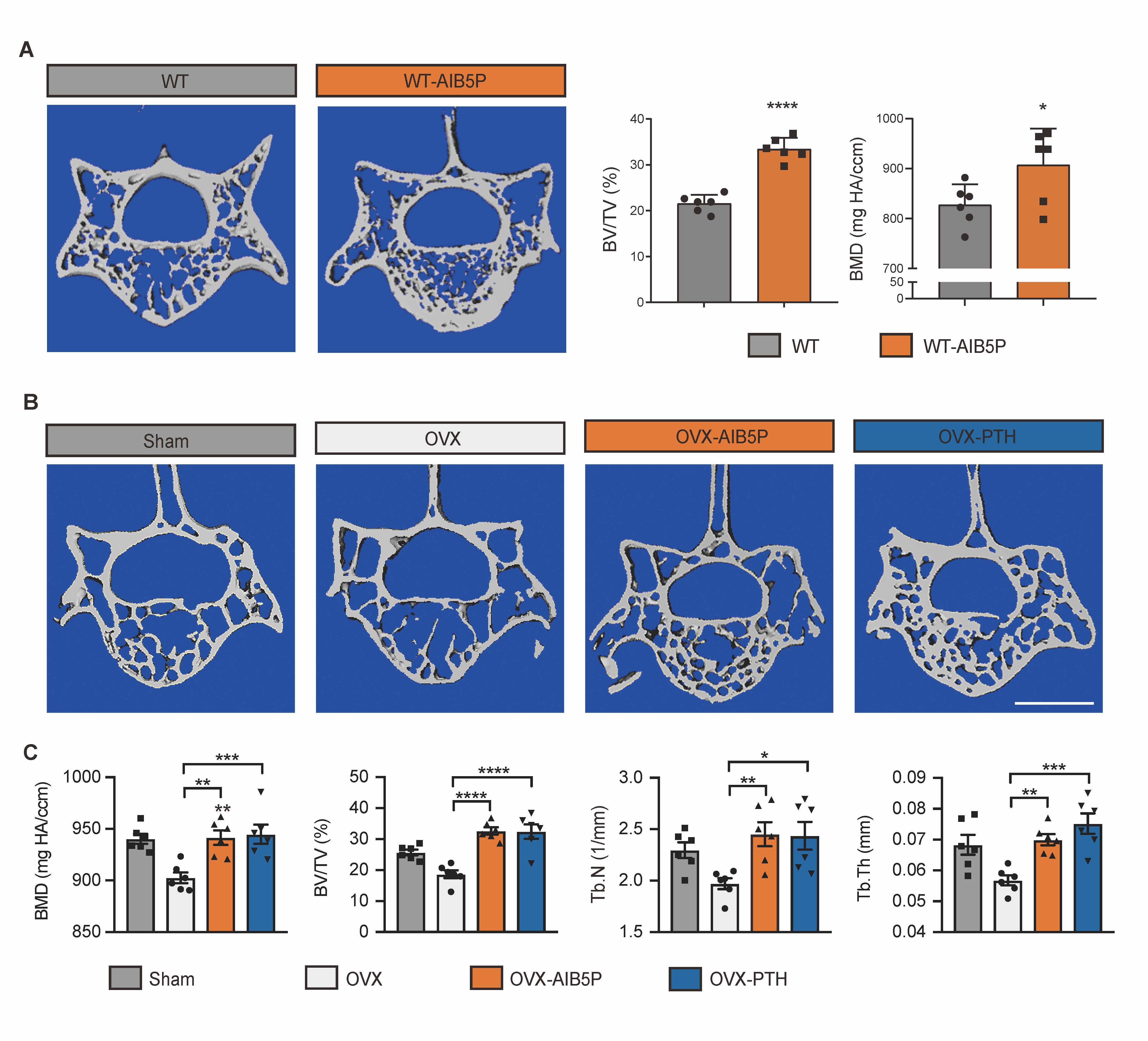

Supplement: Supplementary file 7 — Figure S3 [file 41413_2022_193_MOESM7_ESM.jpg]

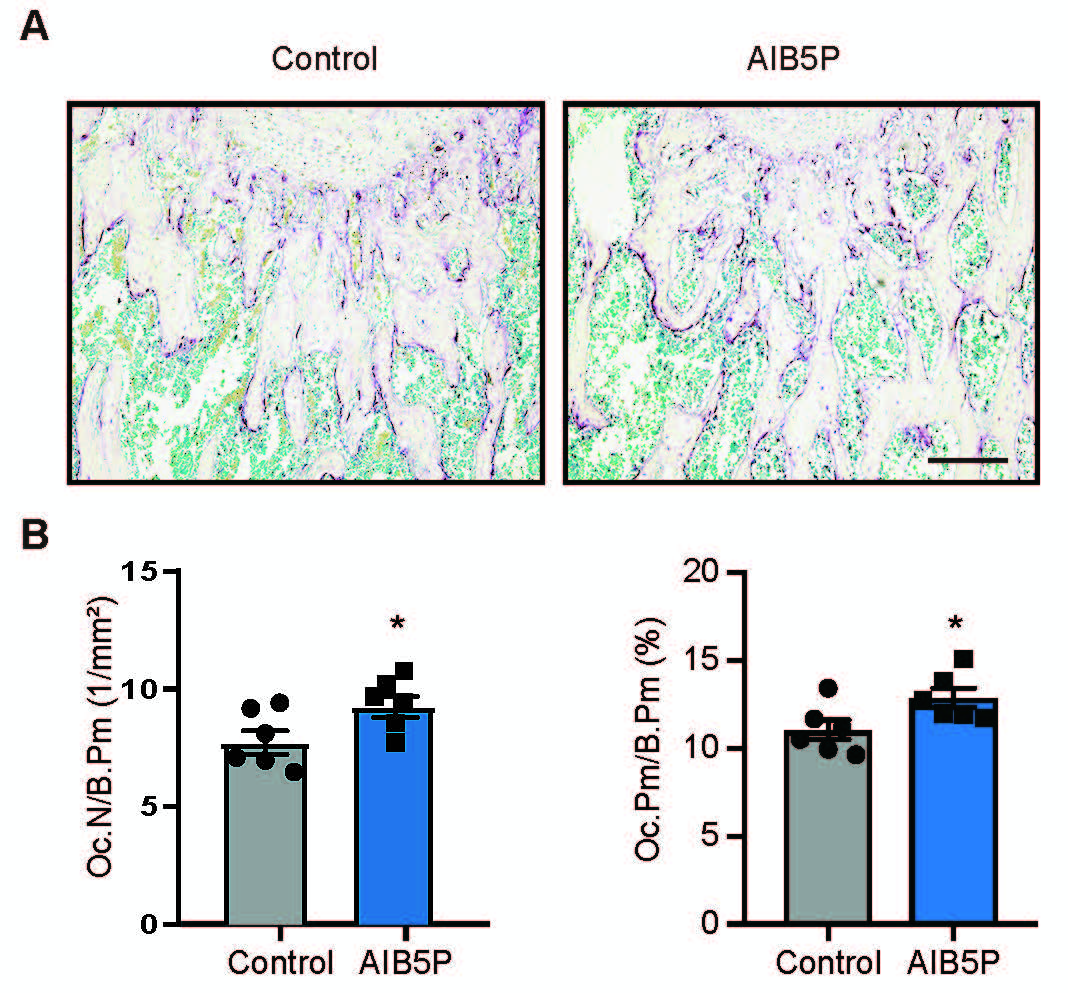

Supplement: Supplementary file 8 — Figure S4 [file 41413_2022_193_MOESM8_ESM.jpg]

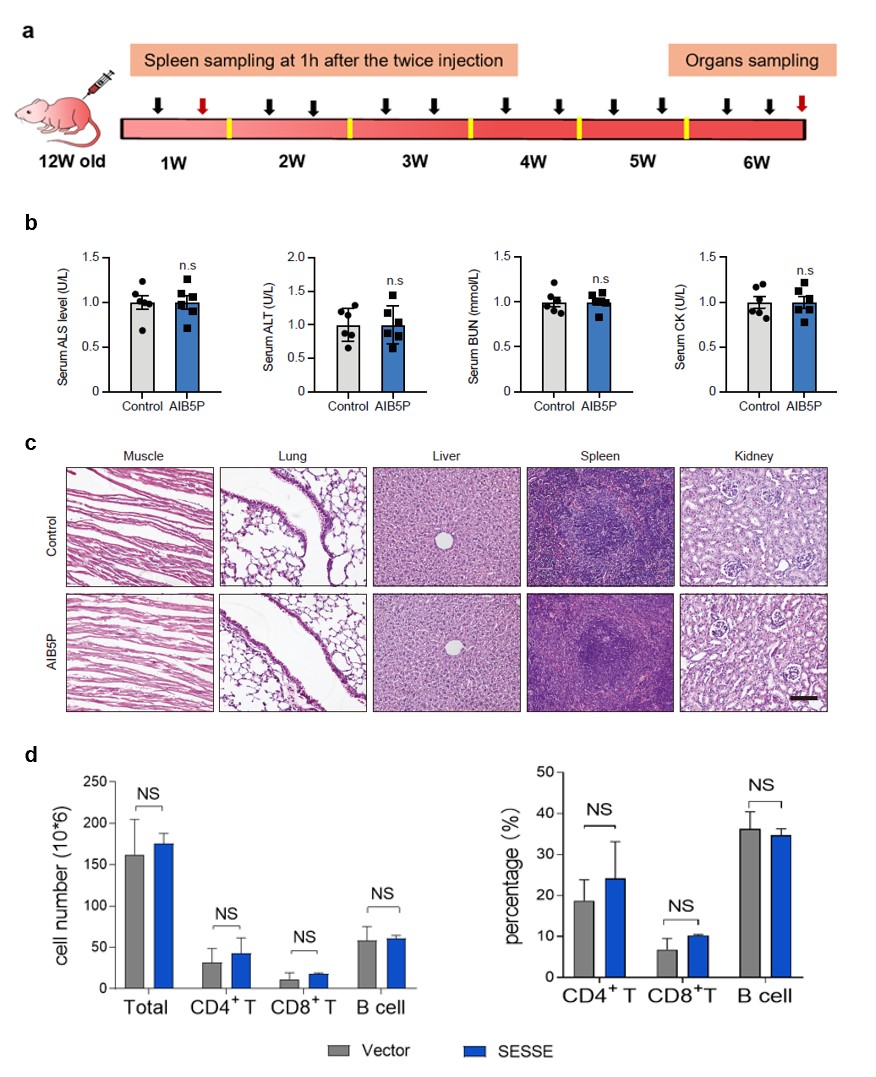

Supplement: Supplementary file 9 — Figure S5 [file 41413_2022_193_MOESM9_ESM.jpg]

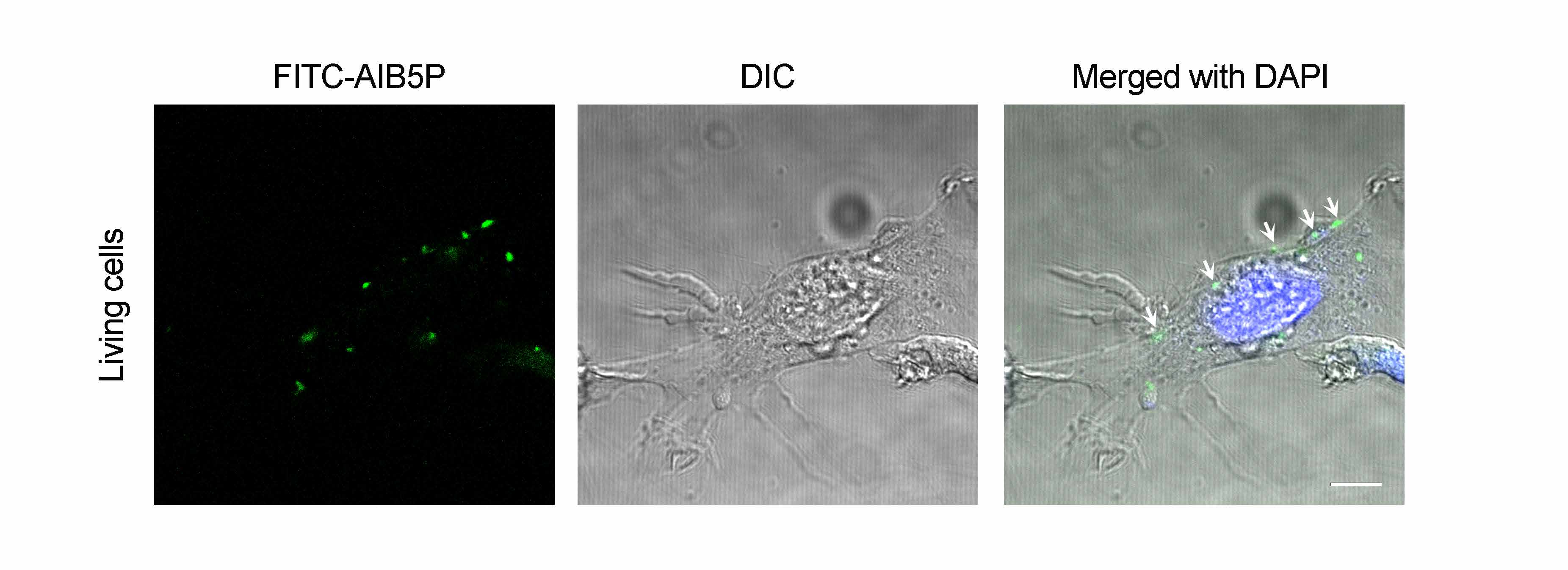

Supplement: Supplementary file 10 — Figure S6 [file 41413_2022_193_MOESM10_ESM.jpg]
